# Late-Onset Neutropenia and Hypogammaglobulinemia After Dose-Adjusted R-EPOCH in Unfavorable Diffuse Large B-Cell Lymphoma

**DOI:** 10.3390/life16091388

**Published:** 2026-08-23

**Authors:** Marko Lucijanić, Rafaela Filipan, Martina Sedinić Lacko, Marija Ivić Čikara, Zdravko Mitrović, Ozren Jakšić

**Affiliations:** 1 Department of Hematology, University Hospital Dubrava, 10000 Zagreb, Croatia; 2 School of Medicine, University of Zagreb, 10000 Zagreb, Croatia; 3 Department of Scientific Research, University Hospital Dubrava, 10000 Zagreb, Croatia

**Keywords:** DA-R-EPOCH, diffuse large B-cell lymphoma, hypogammaglobulinemia, infections, neutropenia, rituximab, body composition, sarcopenia

## Abstract

Background: Late-onset neutropenia (LON) and hypogammaglobulinemia are recognized sequelae of rituximab therapy in B-cell non-Hodgkin lymphoma, and both may leave patients vulnerable to infection once treatment has ended. Methods: Fifty-three patients with newly diagnosed, unfavorable diffuse large B-cell lymphoma (DLBCL) who entered remission on dose-adjusted (DA) R-EPOCH immunochemotherapy were retrospectively evaluated. Neutropenia (absolute neutrophil count < 1.5 × 10^9^/L, CTCAE-graded), hypogammaglobulinemia and infections were registered at treatment completion and 6 and 12 months later. Results: All laboratory parameters changed significantly over time. Most reached their nadir at the end of treatment, whereas the absolute neutrophil count alone reached its lowest value later, at 6 months. Neutropenia, largely mild to moderate, affected 17.6% of patients at treatment completion, 22.4% at 6 months and 7.9% at 12 months. Neutropenia at 6 months was a new event, with no overlap with end-of-treatment neutropenia, and was mostly transient. Hypogammaglobulinemia (IgG < 5 g/L) occurred in 33.3%, 16.7% and 20.0%, respectively; median IgG declined to a nadir at the end of treatment and recovered thereafter, although the deficit tended to persist in the same patients. Post-treatment infections were recorded in 73.6% of patients (mostly respiratory); neutropenia was not associated with infection at any time point, whereas end-of-treatment hypogammaglobulinemia was associated with respiratory infection (*p* = 0.040). The independent predictors of 6-month neutropenia were a greater number of dose-escalated cycles, lower baseline leukocyte count and the absence of on-treatment infection, whereas 6-month hypogammaglobulinemia was predicted by autologous transplantation and the absence of B symptoms. In exploratory body composition analyses, lower baseline psoas muscle mass was associated with end-of-treatment neutropenia (*p* = 0.042) and greater muscle loss with other site (skin and gastrointestinal) infection (*p* = 0.004). Conclusions: After DA-R-EPOCH, LON is a delayed and largely transient event separate from end-of-treatment neutropenia, whereas hypogammaglobulinemia is more persistent and clinically relevant for respiratory infections.

## 1. Introduction

Large B-cell lymphomas constitute nearly a third of all non-Hodgkin lymphomas worldwide, and although most patients present with advanced disease, over 60% are cured by contemporary immunochemotherapy [1]; diagnosis and staging follow established recommendations, including the Lugano classification [2,3,4]. B-cell clearance by rituximab, the first anti-CD20 monoclonal antibody, operates via complement activation, antibody-dependent cellular cytotoxicity, phagocytic clearance and the direct induction of apoptosis [5,6]; combining it with CHOP (R-CHOP) defined the contemporary standard of care [1,7]. For selected patients with high-risk biology, including high-grade B-cell lymphoma carrying MYC together with a BCL2 or BCL6 rearrangement, as well as primary mediastinal disease, the more intensive, pharmacodynamically dosed dose-adjusted R-EPOCH (DA-R-EPOCH) regimen has been used [8,9]. In the randomized CALGB 50303 trial, DA-EPOCH-R conferred no survival benefit over R-CHOP among unselected DLBCL patients while roughly doubling febrile neutropenia and grade 3–4 infection [10] and bearing greater cost [11]; polatuzumab vedotin–R-CHP has since entered the front-line armamentarium [12]. DA-R-EPOCH nonetheless retains a role in defined very-high-risk disease [13].

Two on-target immunological consequences of sustained B-cell depletion are late-onset neutropenia (LON) and hypogammaglobulinemia [14]. LON typically arises one to six months after the last rituximab dose and was first characterized in rituximab-treated lymphoma by Voog and by Chaiwatanatorn et al. [15,16]; the mechanisms proposed for it include disturbed granulopoiesis mediated by stromal-derived factor-1 as B cells reconstitute [17], anti-neutrophil autoantibodies [14], the expansion of cytotoxic large granular lymphocytes [18] and FcγRIIIa-dependent bystander injury [19]. Hypogammaglobulinemia reflects slowed plasma cell renewal and is reported in a substantial minority of treated patients, the estimate varying with cohort and definition [20,21,22,23,24]. Both complications can predispose patients to clinically significant infection, and the systematic post-treatment monitoring of neutrophil counts and immunoglobulin levels has been advocated [14,23].

Host factors may further modify these risks. Sarcopenia is an established adverse prognostic factor in DLBCL and has been linked to greater immunochemotherapy toxicity and to infection [25,26,27,28,29,30]; in this regimen specifically, pronounced muscle loss during DA-R-EPOCH has been linked to poorer clinical outcomes [31]. However, most data on LON and hypogammaglobulinemia derive from R-CHOP-treated cohorts rather than from DA-R-EPOCH, in which prolonged infusional therapy is intensified cycle by cycle according to hematopoietic reserve [8,9,32]. The post-treatment immune–hematological trajectory after DA-R-EPOCH, its relationship to infection, and the influence of body composition therefore remain poorly characterized. We retrospectively examined these outcomes over the first 12 months after DA-R-EPOCH in patients with unfavorable DLBCL.

## 2. Materials and Methods

### 2.1. Study Design and Patient Population

We retrospectively evaluated a single-center, unselected, consecutive cohort of newly diagnosed DLBCL patients who achieved remission after the first-line treatment with DA-R-EPOCH at our institution (University Hospital Dubrava, Zagreb, Croatia) in the period from 2015 to 2019. According to the institutional protocol previously described by the center [32], DA-R-EPOCH was used as primary therapy for DLBCL with unfavorable prognostic features defined as Ki-67 ≥ 80% of tumor cells and/or IPI ≥ 2. Where the disease was of very high risk (Ki-67 ≥ 90% and/or age-adjusted IPI ≥ 2) and comorbidity did not preclude it, consolidation with autologous hematopoietic stem cell transplantation (ASCT) followed. Involved-field radiotherapy was added in selected patients when the treating physician judged it indicated. Exclusion applied to anyone who did not complete the planned course or failed to enter remission, so 53 patients remained for analysis (44 in complete and 9 in partial remission).

### 2.2. Treatment Protocol

The regimen reproduced the originally published DA-EPOCH schedule [8]. Etoposide (50 mg/m^2^/day), vincristine (0.4 mg/m^2^/day) and doxorubicin (10 mg/m^2^/day) were each delivered as a continuous infusion across days 1–4. Prednisone 60 mg/m^2^/day was taken orally on days 1–5 or replaced by an equipotent intravenous methylprednisolone dose. Cyclophosphamide 750 mg/m^2^/day was administered on day 5. Rituximab 375 mg/m^2^ was given on day 1 of every cycle. Six to eight cycles were planned. Cycles recurred at 21-day intervals under obligatory myeloid support, either filgrastim 5 µg/kg daily from day 6 to neutrophil recovery or a single 6 mg dose of pegfilgrastim on day 6. Whether cotrimoxazole, acyclovir and fluconazole prophylaxis was added rested with the treating physician. Interim response was assessed by computed tomography (CT) after four cycles: patients in complete remission (CR) received two further cycles (total of six) and those in partial remission (PR) four further cycles (total of eight), with final response assessment by CT at treatment completion. The post-treatment use of G-CSF and intravenous immunoglobulin (IVIG) was not institutionally protocolized and followed individual clinical indications.

### 2.3. Laboratory and Body Composition Measurements and Definitions

Four assessment points were used: at diagnosis, on the completion of treatment, and at the 6- and 12-month post-treatment follow-up visits. At each, the complete blood count was recorded—total leukocytes (Leu); absolute neutrophil (ANC), lymphocyte (ALC) and monocyte (AMC) counts; hemoglobin (Hgb) and platelets (Plt)—together with serum IgG and C-reactive protein (CRP) if available. ANC reduction was categorized by CTCAE as mild (grade 2; ANC 1.0–1.5 × 10^9^/L), moderate (grade 3; ANC 0.5–1.0 × 10^9^/L) or severe (grade 4; ANC < 0.5 × 10^9^/L). An IgG concentration under 5 g/L denoted hypogammaglobulinemia, with additional strata drawn at 4 g/L and 2 g/L. Post-treatment infections were abstracted from clinical records and categorized by site as respiratory, urinary or other (predominantly skin and gastrointestinal). Skeletal muscle was quantified on staging and restaging CT from the bilateral psoas muscle area, which was indexed to patient height; change during therapy was expressed as the follow-up-to-baseline psoas ratio, and sarcopenia was additionally assigned using pre-specified sex-specific thresholds [26].

### 2.4. Statistical Analysis

The distribution of numerical variables was checked with the Shapiro–Wilk test. Because departure from normality prevailed for most variables, continuous data are summarized as the median with interquartile range (IQR) and compared between groups by the Mann–Whitney U test; frequencies are reported as counts with percentages and compared by Fisher’s exact test or χ^2^, as appropriate. The Spearman coefficient was used for correlation. Serial within-patient changes were examined pairwise by the Wilcoxon signed-rank test for continuous measures and by the McNemar test for binary measures, whereas change across all time points was tested globally by the Friedman test. Variables independently predicting binary endpoints were identified by multivariable logistic regression. Significance was set at a two-sided *p* < 0.05. Computations were performed in MedCalc v23.2.1 (MedCalc Software, Ostend, Belgium) and Python 3.11. Given the exploratory design and limited sample, univariate comparisons were not corrected for multiplicity and are regarded as hypothesis-generating; no validation cohort was available.

### 2.5. Ethics

Institutional approval was granted by the Ethics Committee of University Hospital Dubrava under no. 2025/0424-5, and this work adhered throughout to the principles of the Declaration of Helsinki. Because the analysis was entirely retrospective and drew only on de-identified data generated in routine care, the Committee waived the need to obtain written informed consent.

## 3. Results

### 3.1. Patient, Disease, and Treatment Characteristics

Fifty-three patients (28 men, 52.8%; 25 women, 47.2%; median age 64 years, IQR 51–72) were analyzed. The cohort had advanced, high-risk disease: 62.3% had Ann Arbor stage III–IV, 60.4% had elevated LDH, the median Ki-67 was 80% (IQR 60–90), 52.8% had more than one extranodal site and 35.8% had bulky disease; the median IPI and R-IPI were both 3. A median of six DA-R-EPOCH cycles was delivered (IQR 6–7), of which a median of two cycles were dose-escalated (IQR 1–3) and zero dose-reduced (IQR 0–1). Fourteen patients (26.4%) were consolidated with ASCT, and four (7.5%) received involved-field radiotherapy. Forty-four patients (83.0%) achieved complete remission and nine (17.0%) partial remission. Infection during DA-R-EPOCH treatment was documented in 26 patients (49.1%) and febrile neutropenia in 23 of 50 evaluable patients (46.0%) (Table 1). In the post-treatment period, a total of six patients received G-CSF—one with neutropenia at the end of treatment, four up to 6 months, and one up to 12 months post-treatment—and IVIG was administered to two patients.

### 3.2. Dynamics of Laboratory Parameters over Time

**Figure 1:**
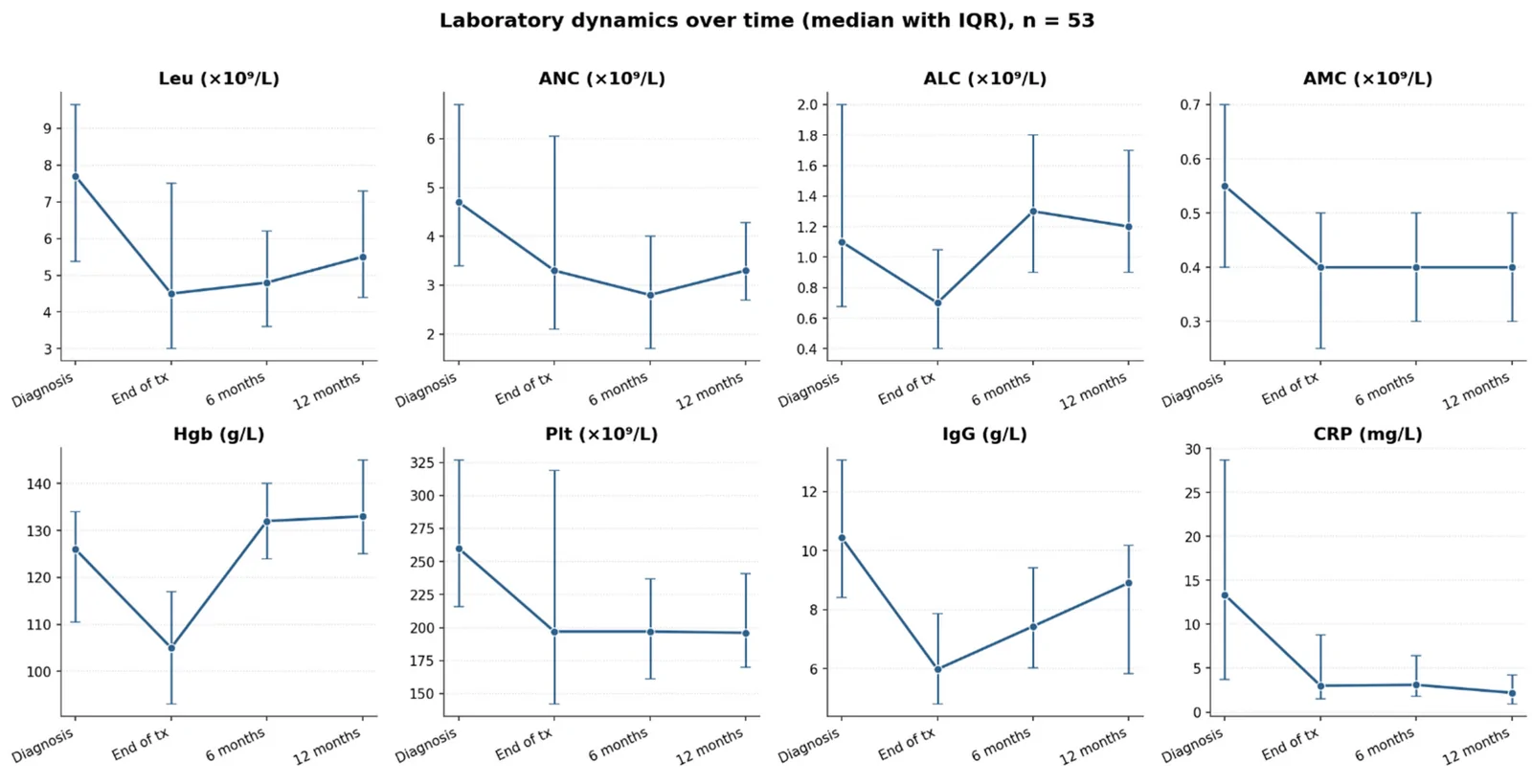
Laboratory dynamics over time (median, IQR; n = 53).

All analyzed parameters changed significantly over time (Friedman test *p* < 0.05 for all). Total leukocytes, the absolute lymphocyte count and IgG were the lowest at the end of treatment and then recovered progressively, whereas the ANC reached its nadir later, at 6 months and recovered only partially by 12 months. Hemoglobin followed a similar early nadir pattern, falling to its lowest value at the end of treatment before recovering to slightly above its baseline by 6–12 months, whereas platelets declined by the end of treatment and remained modestly below baseline thereafter. The absolute monocyte count remained below baseline at every post-treatment time point, and CRP fell progressively from diagnosis to 12 months (Table 2, Figure 1).

**Figure 2:**
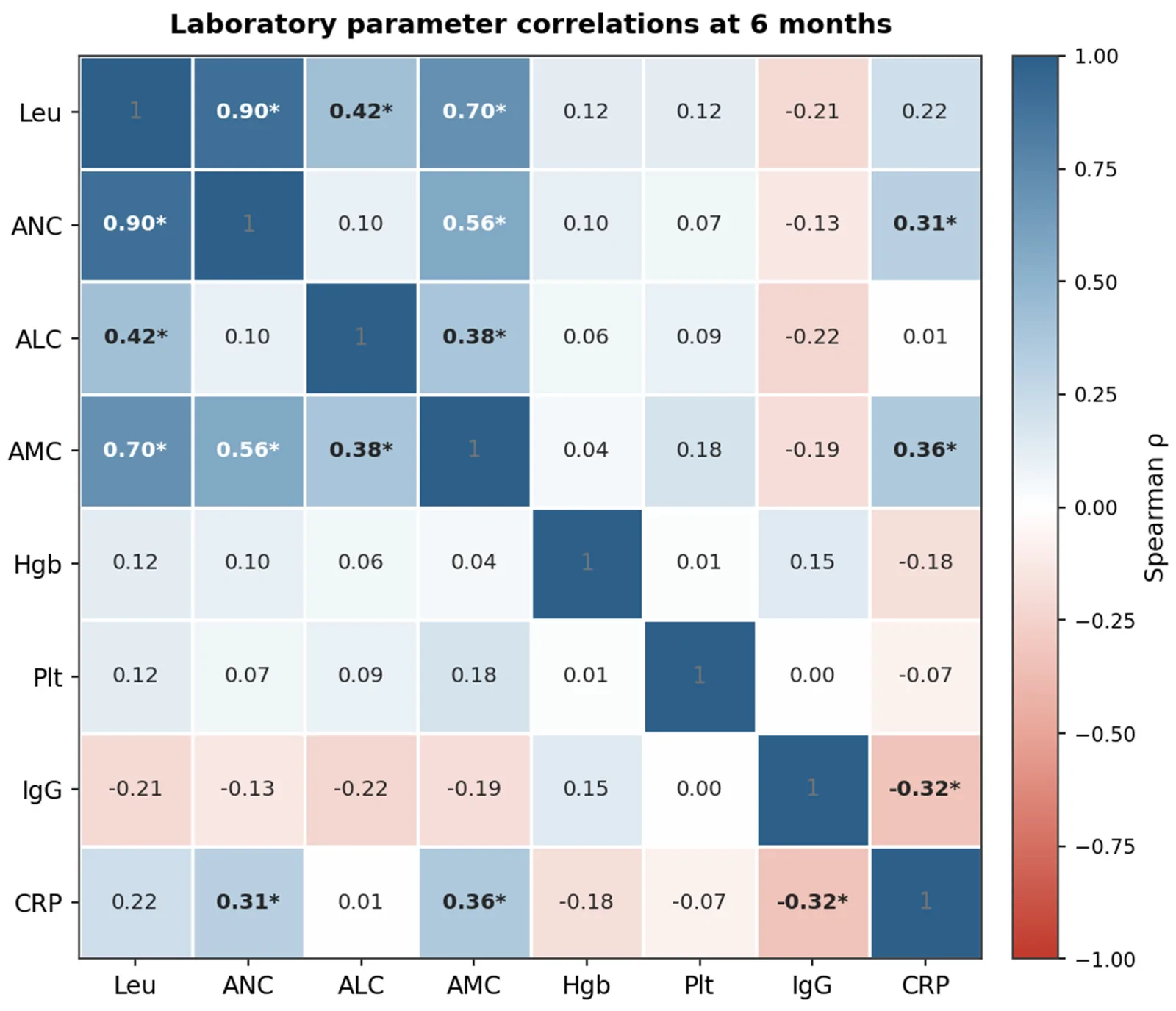
Spearman correlation matrix among laboratory parameters at 6-month time point. Asterisks (∗) and bold formatting mark correlations that reach statistical significance (*p* < 0.05).

At the 6-month time point, the myeloid counts were strongly inter-correlated (Leu–ANC), the lymphoid and monocyte counts correlated moderately with the leukocyte count, and IgG was weakly and inversely related to the myeloid counts and to CRP; hemoglobin and platelets were largely independent of the other parameters (Figure 2).

### 3.3. Incidence, Trajectory and Predictors of Neutropenia

**Figure 3:**
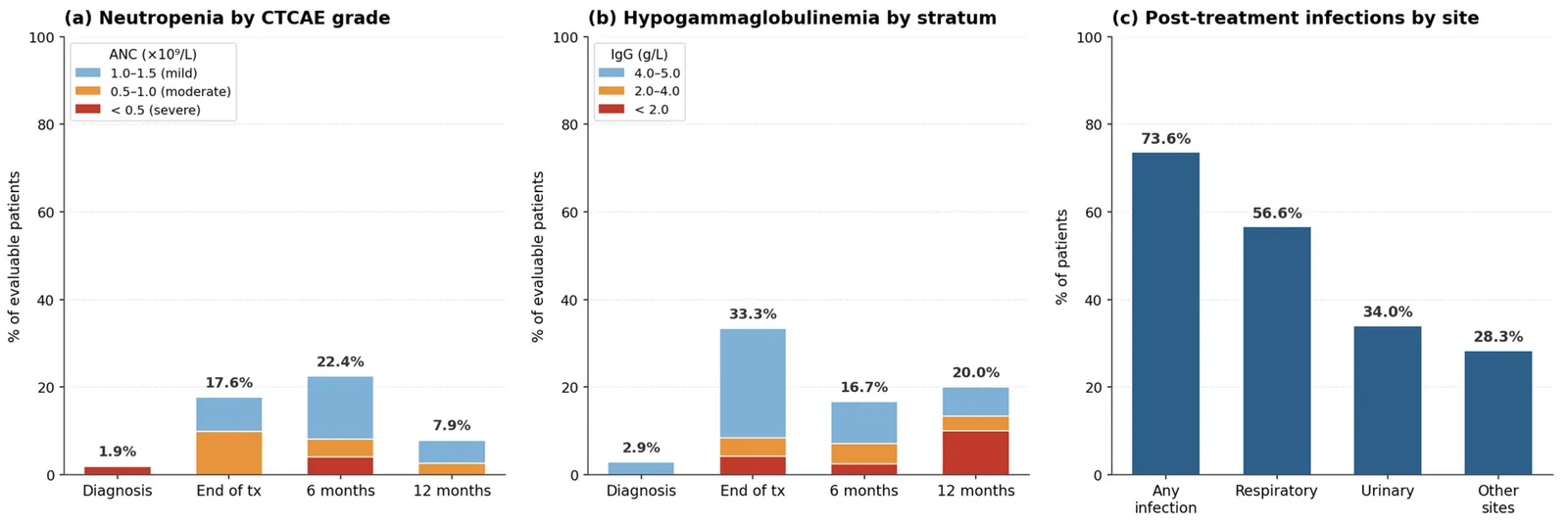
(**a**) Neutropenia by CTCAE grade, (**b**) hypogammaglobulinemia by stratum, and (**c**) post-treatment infections by site, expressed as percentages of evaluable patients at each time point.

**Figure 4:**
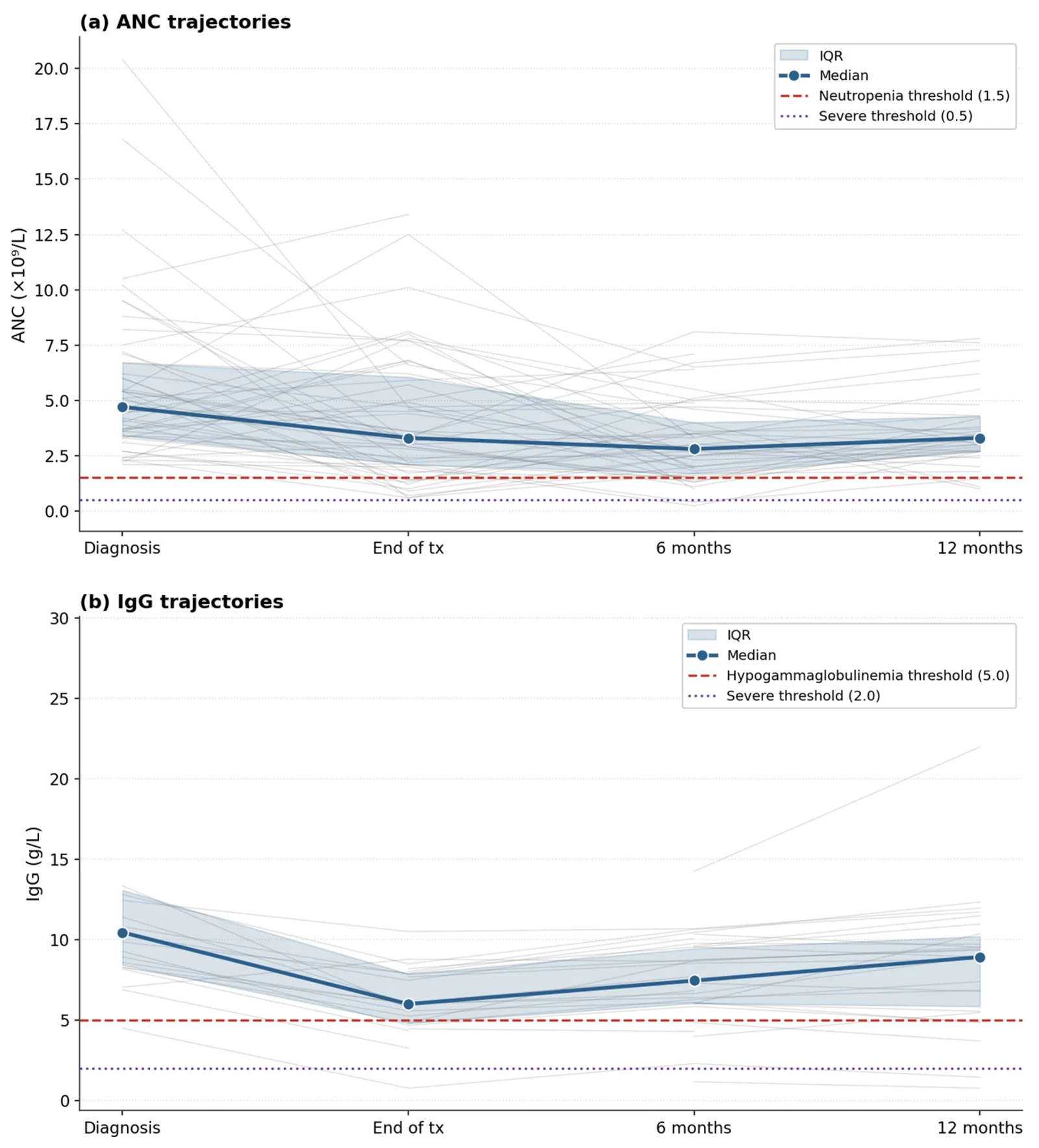
Individual-patient trajectories (gray) with median and interquartile range. (**a**) Absolute neutrophil count (ANC), with neutropenia (1.5 × 10^9^/L) and severe (0.5 × 10^9^/L) thresholds; median reaches its nadir at 6 months. (**b**) Serum IgG, with hypogammaglobulinemia (5 g/L) and severe (2 g/L) thresholds; median is lowest at end of treatment and recovers thereafter.

Neutropenia of any grade was present in 9 patients (17.6%) at the end of treatment (4 mild, 5 moderate), in 11 patients (22.4%) at 6 months (7 mild, 2 moderate, 2 severe) and in 3 patients (7.9%) at 12 months (Figure 3a). Compared with diagnosis, the proportion of neutropenic patients was significantly greater at the end of treatment and at 6 months (both *p* < 0.05), whereas the post-treatment time points did not differ significantly from one another. The individual-patient ANC trajectories, with the median reaching its nadir at 6 months, are shown in Figure 4a. No patient at the end of treatment remained neutropenic at 6 months, identifying 6-month neutropenia as a new event rather than persistent on-treatment toxicity; most patients with 6-month neutropenia (10 of 11) recovered by 12 months.

In univariate analysis, neutropenia at the end of treatment was associated with lower body weight (*p* = 0.009), lower height (*p* = 0.021) and smaller body surface area (*p* = 0.005); older age (*p* = 0.055) and female sex (*p* = 0.057) were of borderline significance. Neutropenia at 6 months was associated with younger age (*p* = 0.001), lower IPI (*p* = 0.016) and R-IPI (*p* = 0.024), a greater number of dose-escalated cycles (*p* = 0.013), the use of ASCT (*p* = 0.017), fewer on-treatment infections (*p* = 0.043), lower leukocyte count at diagnosis (*p* = 0.023) and lower CRP at the 6-month visit (*p* = 0.040); sex was not associated at 6 or 12 months (both *p* > 0.05) nor was age at 12 months (*p* = 0.61). In multivariate logistic regression, the independent predictors of 6-month neutropenia were a greater number of dose-escalated cycles (odds ratio [OR] 3.62, 95% confidence interval [CI] 1.41–9.32; *p* = 0.075), lower leukocyte count at diagnosis (OR 0.63, 95% CI 0.41–0.98; *p* = 0.041) and the absence of on-treatment infection (OR 18.25, 95% CI 1.43–231.8; *p* = 0.025). No independent predictors were identified at the end of treatment or at 12 months.

### 3.4. Incidence, Trajectory and Predictors of Hypogammaglobulinemia

An IgG below 5 g/L was documented in 1 of 35 evaluable patients (2.9%) at diagnosis, in 8 of 24 (33.3%) at the end of treatment, in 7 of 42 (16.7%) at 6 months and in 6 of 30 (20.0%) at 12 months (Figure 3b); deeper deficits (IgG < 2 g/L) were the most frequent at 12 months (3 of 30, 10.0%). The individual-patient IgG trajectories, with the median lowest value at the end of treatment and recovering thereafter, are shown in Figure 4b. In contrast to neutropenia, hypogammaglobulinemia tended to involve the same patients over time: of 19 patients with paired data, 2 (10.5%) were hypogammaglobulinemic both at the end of treatment and at 6 months, and of 16 with paired data, 3 (18.8%) were hypogammaglobulinemic at both the end of treatment and 12 months.

Hypogammaglobulinemia at the end of treatment was associated with partial rather than complete remission (*p* = 0.013), lower IgG at diagnosis (*p* = 0.029) and higher leukocyte and ANC at the end of treatment (*p* = 0.037 and *p* = 0.004). Hypogammaglobulinemia at 6 months was associated with the absence of B symptoms (*p* = 0.038), lower IgG at the end of treatment (*p* = 0.024) and ASCT (*p* = 0.023). Hypogammaglobulinemia at 12 months was associated with the absence of B symptoms (*p* = 0.025), partial remission (*p* = 0.005) and lower IgG at every prior time point (all *p* ≤ 0.014). In multivariate logistic regression, the independent predictors of 6-month hypogammaglobulinemia were ASCT (OR 17.99, 95% CI 1.55–207.7; *p* = 0.021) and the absence of B symptoms (OR 0.04, 95% CI 0.00–0.77; *p* = 0.032).

### 3.5. Post-Treatment Infections

During the 12 months after the end of treatment, 39 of 53 patients (73.6%) had at least one documented infection: 30 (56.6%) respiratory, 18 (34.0%) urinary and 15 (28.3%) at another site (Figure 3c). The occurrence of any infection was associated with the absence of B symptoms at diagnosis (*p* = 0.010) and a lower proliferation index (Ki-67; *p* = 0.048), with a borderline association for lower IgG at diagnosis (*p* = 0.099). Neutropenia at none of the three post-treatment time points was associated with the infection of any site (all *p* > 0.05), whereas hypogammaglobulinemia at treatment completion carried a higher frequency of respiratory infection (*p* = 0.040), without any association for the total infection rate or for other infection sites.

### 3.6. Body Composition and Post-Treatment Outcomes

Lower baseline psoas muscle area was associated with neutropenia at the end of treatment (*p* = 0.042) but not with neutropenia at 6 or 12 months, with hypogammaglobulinemia at any time point, or with any infection category (all *p* > 0.05). Greater treatment-associated psoas muscle loss was associated with infection at non-respiratory, non-urinary (other) sites (*p* = 0.004) and showed a non-significant trend toward any infection (*p* = 0.065). No muscle metric was associated with IgG at any time point (all *p* > 0.1).

## 4. Discussion

In this retrospective series from a single institution, we mapped how blood counts and IgG levels evolve once DA-R-EPOCH for unfavorable DLBCL has been completed. Three findings predominate. First, the absolute neutrophil count reached its nadir at 6 months, and no patient at the end of treatment remained neutropenic at 6 months, identifying LON as a delayed event distinct from on-treatment cytopenia. Second, median IgG declined to a nadir at the end of treatment and recovered thereafter, yet hypogammaglobulinemia tended to persist in the same patients. Third, end-of-treatment hypogammaglobulinemia, rather than neutropenia, was associated with post-treatment respiratory infection.

The 6-month timing of LON is concordant with the prospective National Cancer Institute DA-EPOCH cohorts, in which LON followed treatment by a median of approximately six months [17,33], and with the early rituximab-treated lymphoma series of Voog and Chaiwatanatorn [15,16]. Reported LON incidences vary widely with the definition and regimen used: among DLBCL patients given rituximab-based first-line therapy, LON of any grade has been reported in up to 26.9% (severe LON in 7.5%) of patients [34], whereas the randomized GOYA trial recorded LON in 4.9% of R-CHOP-treated and 8.7% of obinutuzumab–CHOP-treated patients [35], and a rate of approximately 8% was observed after DA-EPOCH-based therapy [17]. The proposed mechanisms of LON, namely perturbed granulopoiesis during B-cell reconstitution [17], anti-neutrophil autoantibodies [14], large granular lymphocyte expansion [18] and FcγRIIIa-dependent injury [19], are not mutually exclusive and may also be reflected in the independent predictors observed here. Greater dose intensification plausibly produces deeper and more sustained progenitor injury that manifests as a delayed neutrophil nadir, while a lower baseline leukocyte count may indicate limited bone marrow reserve. The association of LON with the absence of on-treatment infection is biologically coherent, since infection is a potent stimulus of granulopoiesis, in which cytokine signaling expands myeloid output [36]; patients without infection may therefore receive less granulopoietic stimulation and, having tolerated more dose-dense therapy without interruption, present a more pronounced delayed nadir.

The incidence of hypogammaglobulinemia (33.3%, 16.7% and 20.0% at end of treatment and 6 and 12 months, respectively) is consistent with rituximab-treated lymphoma series, in which new hypogammaglobulinemia affects roughly one-quarter to one-half of patients, and a minority require intravenous immunoglobulin (IVIG) treatment [20,22,23,24]. Its persistence in the same patients is in line with prolonged B-cell depletion and slow humoral reconstitution. The independent association of 6-month hypogammaglobulinemia with autologous transplantation is mechanistically plausible: high-dose conditioning ablates residual B cells and long-lived plasma cells, while the characteristically slow reconstitution of B cells and immunoglobulin after transplantation—further delayed in DLBCL by prior anti-CD20 therapy, with CD19+ recovery reportedly subnormal for at least 18 months—prolongs the humoral deficit [37]. The association with the absence of B symptoms is less readily explained. It may reflect reduced lymphoma-driven immune stimulation, but the direction of the association could also be reversed: patients predisposed to humoral immune deficiency may be intrinsically less able to mount the systemic cytokine response that generates B symptoms, rather than the absence of B symptoms promoting hypogammaglobulinemia. A comparable interpretation may apply to the univariate association of post-treatment infection with the absence of B symptoms and a lower proliferation index, which likely mark a less inflammatory, biologically less aggressive disease phenotype and a host with a constitutionally weaker cytokine response that both blunts B-symptom generation and may impair infection control; given their borderline significance and the number of comparisons, these associations are hypothesis-generating. That end-of-treatment hypogammaglobulinemia, rather than neutropenia, tracked with respiratory infection is consistent with the humoral basis of mucosal respiratory defense and with the predominance of respiratory infection in this cohort (56.6%) [14,21,23]. Systematic post-treatment IgG monitoring, with IVIG treatment for symptomatic patients, is therefore reasonable [38].

Body composition contributed a host-related signal: lower baseline psoas muscle area was associated with end-of-treatment neutropenia and greater muscle loss with non-respiratory infection, consistent with reports linking low skeletal muscle to immunochemotherapy toxicity and infection in DLBCL, including after DA-R-EPOCH [27,28,31]. Similarly, the associations of end-of-treatment neutropenia with lower body weight, height, and body surface area, which were present in univariate analysis but did not persist after multivariable adjustment, likely reflect poorer nutritional status and limited hematopoietic reserve. No muscle metric was associated with IgG, suggesting that the humoral deficit is driven primarily by B-cell depletion rather than by nutritional status.

We would like to highlight several limitations of the current study. First, its retrospective nature, single-institution context, and modest cohort size together with the attrition of laboratory data on specific time points (especially for IgG) constrain both statistical power and external validity. However, patients with and without evaluable 12-month IgG did not differ significantly in any evaluated characteristic, with only a non-significant trend toward lower end-of-treatment IgG among those with missing measurements. With few events and correspondingly wide confidence intervals, the derived estimates and associations are best read as exploratory and hypothesis-generating. Second, supportive care factors that directly affect neutrophil and immunoglobulin recovery, as well as the management of post-treatment infections, were not institutionally protocolized and were addressed on an individual basis. Post-treatment infections were mostly defined clinically and radiologically, with infrequent microbiological confirmation, precluding a pathogen-specific analysis. Third, body composition was estimated from the psoas muscle as a surrogate for total skeletal muscle mass. Accordingly, the reported associations are hypothesis-generating and warrant prospective, multicenter validation.

## 5. Conclusions

In patients with unfavorable DLBCL who achieve remission after DA-R-EPOCH, late-onset neutropenia and hypogammaglobulinemia follow distinct courses. LON is a delayed, largely transient event peaking at 6 months and is independently predicted by greater dose intensification, lower baseline leukocyte count and the absence of on-treatment infection. Although median IgG recovers after an end-of-treatment nadir, hypogammaglobulinemia tends to persist in individual patients and, when present at the end of treatment, is the principal correlate of post-treatment respiratory infection. These findings support the systematic post-treatment monitoring of both the neutrophil count and serum IgG after DA-R-EPOCH, with IVIG treatment considered for symptomatic hypogammaglobulinemia [38]. Prospective, multicenter validation is warranted.

## Figures and Tables

**Table 1:** Baseline patient, disease, and treatment characteristics (n = 53).

Characteristic	Value (n = 53)
Age, years, median (IQR)	64 (51–72)
Male/female	28 (52.8%)/25 (47.2%)
BMI, kg/m^2^, median (IQR)	28.0 (25.0–30.3)
Body surface area, m^2^, median (IQR)	1.89 (1.77–2.05)
Ann Arbor I/II/III/IV	1 (1.9%)/19 (35.8%)/13 (24.5%)/20 (37.7%)
Elevated LDH	32 (60.4%)
ECOG, median (IQR)	1 (1–1)
Ki-67, %, median (IQR)	80 (60–90)
>1 extranodal site	28 (52.8%)
Bulky disease	19 (35.8%)
B symptoms	26 (49.1%)
IPI low/low-int/high-int/high	13 (24.5%)/10 (18.9%)/13 (24.5%)/17 (32.1%)
R-IPI very good/good/poor	3 (5.7%)/21 (39.6%)/29 (54.7%)
DA-R-EPOCH cycles, median (IQR)	6 (6–7)
Dose-escalated cycles, median (IQR)	2 (1–3)
Dose-reduced cycles, median (IQR)	0 (0–1)
Infection during treatment	26 (49.1%)
Febrile neutropenia during treatment	23/50 (46.0%)
Autologous stem cell transplant	14 (26.4%)
Radiotherapy	4 (7.5%)
Response: Complete/partial remission	44 (83.0%)/9 (17.0%)

Counts with percentages (categorical variables) and medians with interquartile range (continuous variables) were used for data summarization. Abbreviations: BMI, body mass index; DA-R-EPOCH, dose-adjusted rituximab, etoposide, prednisone, vincristine, cyclophosphamide and doxorubicin; ECOG, Eastern Cooperative Oncology Group performance status; int, intermediate; IPI, International Prognostic Index; IQR, interquartile range; Ki-67, proliferation index; LDH, lactate dehydrogenase; R-IPI, Revised International Prognostic Index.

**Table 2:** Laboratory dynamics: median (IQR) at each time point.

Parameter	Diagnosis	End of tx	6 Months	12 Months
Leu (×10^9^/L)	7.7 (5.4–9.7)	4.5 (3.0–7.5)	4.8 (3.6–6.2)	5.5 (4.4–7.3)
ANC (×10^9^/L)	4.7 (3.4–6.7)	3.3 (2.1–6.1)	2.8 (1.7–4.0)	3.3 (2.7–4.3)
ALC (×10^9^/L)	1.1 (0.7–2.0)	0.7 (0.4–1.1)	1.3 (0.9–1.8)	1.2 (0.9–1.7)
AMC (×10^9^/L)	0.55 (0.4–0.7)	0.4 (0.25–0.5)	0.4 (0.3–0.5)	0.4 (0.3–0.5)
Hgb (g/L)	126 (111–134)	105 (93–117)	132 (124–140)	133 (125–145)
Plt (×10^9^/L)	260 (216–327)	197 (142–319)	197 (161–237)	196 (170–241)
IgG (g/L)	10.4 (8.4–13.1)	6.0 (4.8–7.9)	7.4 (6.0–9.4)	8.9 (5.8–10.2)
CRP (mg/L)	13.3 (3.7–28.7)	3.0 (1.5–8.8)	3.1 (1.8–6.4)	2.2 (0.9–4.2)

Data are summarized as medians with interquartile ranges. Abbreviations: ALC, absolute lymphocyte count; AMC, absolute monocyte count; ANC, absolute neutrophil count; CRP, C-reactive protein; Hgb, hemoglobin; IgG, immunoglobulin G; IQR, interquartile range; Leu, total leukocyte count; Plt, platelets; tx, treatment.

## Data Availability

The datasets underlying this report may be obtained from the corresponding author upon reasonable request; they cannot be deposited publicly because institutional rules restrict the sharing of patient-level clinical records.

## References

[1] Sehn L.H., Salles G. (2021). Diffuse large B-cell lymphoma. N. Engl. J. Med..

[2] Mey U., Hitz F., Lohri A., Pederiva S., Taverna C., Tzankov A., Meier O., Yeow K., Renner C. (2012). Diagnosis and treatment of diffuse large B-cell lymphoma. Swiss Med. Wkly..

[3] Tilly H., Gomes da Silva M., Vitolo U., Jack A., Meignan M., Lopez-Guillermo A., Walewski J., André M., Johnson P.W., Pfreundschuh M. (2015). Diffuse large B-cell lymphoma (DLBCL): ESMO Clinical Practice Guidelines for diagnosis, treatment and follow-up. Ann. Oncol..

[4] Cheson B.D., Fisher R.I., Barrington S.F., Cavalli F., Schwartz L.H., Zucca E., Lister T.A. (2014). Recommendations for initial evaluation, staging, and response assessment of Hodgkin and non-Hodgkin lymphoma: The Lugano classification. J. Clin. Oncol..

[5] Smith M.R. (2003). Rituximab (monoclonal anti-CD20 antibody): Mechanisms of action and resistance. Oncogene.

[6] Klein C., Lammens A., Schäfer W., Georges G., Schwaiger M., Mössner E., Hopfner K.P., Umaña P., Niederfellner G. (2013). Epitope interactions of monoclonal antibodies targeting CD20 and their relationship to functional properties. mAbs.

[7] Coiffier B., Lepage E., Brière J., Herbrecht R., Tilly H., Bouabdallah R., Morel P., Van Den Neste E., Salles G., Gaulard P. (2002). CHOP chemotherapy plus rituximab compared with CHOP alone in elderly patients with diffuse large-B-cell lymphoma. N. Engl. J. Med..

[8] Wilson W.H., Grossbard M.L., Pittaluga S., Cole D., Pearson D., Drbohlav N., Steinberg S.M., Little R.F., Janik J., Gutierrez M. (2002). Dose-adjusted EPOCH chemotherapy for untreated large B-cell lymphomas: A pharmacodynamic approach with high efficacy. Blood.

[9] Major A., Smith S.M. (2021). DA-R-EPOCH vs R-CHOP in DLBCL: How do we choose?. Clin. Adv. Hematol. Oncol..

[10] Bartlett N.L., Wilson W.H., Jung S.H., Hsi E.D., Maurer M.J., Pederson L.D., Polley M.C., Pitcher B.N., Cheson B.D., Kahl B.S. (2019). Dose-adjusted EPOCH-R compared with R-CHOP as frontline therapy for diffuse large B-cell lymphoma: Clinical outcomes of the phase III Intergroup Trial Alliance/CALGB 50303. J. Clin. Oncol..

[11] Dholaria B., Vanegas Y.A.M., Diehl N., Spaulding A.C., Visscher S., Tun H.W., Ailawadhi S., Vishnu P. (2020). Cost analysis of R-CHOP versus dose-adjusted R-EPOCH in treatment of diffuse large B-cell lymphoma with high-risk features. Clin. Hematol. Int..

[12] Tilly H., Morschhauser F., Sehn L.H., Friedberg J.W., Trněný M., Sharman J.P., Herbaux C., Burke J.M., Matasar M., Rai S. (2022). Polatuzumab vedotin in previously untreated diffuse large B-cell lymphoma. N. Engl. J. Med..

[13] Alduaij W., Sehn L.H., Champagne J.N., Collinge B., Ben-Neriah S., Jiang A., Hilton L.K., Boyle M., Meissner B., Slack G.W. (2026). Population-wide introduction of dose-adjusted EPOCH-R in high-grade B-cell lymphoma with MYC/BCL2 rearrangements, DLBCL morphology. Blood Adv..

[14] Athni T.S., Barmettler S. (2023). Hypogammaglobulinemia, late-onset neutropenia, and infections following rituximab. Ann. Allergy Asthma Immunol..

[15] Voog E., Morschhauser F., Solal-Céligny P. (2003). Neutropenia in patients treated with rituximab. N. Engl. J. Med..

[16] Chaiwatanatorn K., Lee N., Grigg A., Filshie R., Firkin F. (2003). Delayed-onset neutropenia associated with rituximab therapy. Br. J. Haematol..

[17] Dunleavy K., Hakim F., Kim H.K., Janik J.E., Grant N., Nakayama T., White T., Wright G., Kwak L., Gress R. (2005). B-cell recovery following rituximab-based therapy is associated with perturbations in stromal derived factor-1 and granulocyte homeostasis. Blood.

[18] Papadaki T., Stamatopoulos K., Stavroyianni N., Paterakis G., Phisphis M., Stefanoudaki-Sofianatou K. (2002). Evidence for T-large granular lymphocyte-mediated neutropenia in Rituximab-treated lymphoma patients: Report of two cases. Leuk. Res..

[19] Weng W.K., Negrin R.S., Lavori P., Horning S.J. (2010). Immunoglobulin G Fc receptor FcγRIIIa 158 V/F polymorphism correlates with rituximab-induced neutropenia after autologous transplantation in patients with non-Hodgkin’s lymphoma. J. Clin. Oncol..

[20] Makatsori M., Kiani-Alikhan S., Manson A.L., Verma N., Leandro M., Gurugama N.P., Longhurst H.J., Grigoriadou S., Buckland M., Kanfer E. (2014). Hypogammaglobulinaemia after rituximab treatment—Incidence and outcomes. QJM.

[21] Gkrania-Klotsas E., Kumararatne D.S. (2021). Serious infectious complications after rituximab therapy in patients with autoimmunity: Is this the final word?. Clin. Infect. Dis..

[22] Casulo C., Maragulia J., Zelenetz A.D. (2013). Incidence of hypogammaglobulinemia in patients receiving rituximab and the use of intravenous immunoglobulin for recurrent infections. Clin. Lymphoma Myeloma Leuk..

[23] Barmettler S., Ong M.S., Farmer J.R., Choi H., Walter J. (2018). Association of immunoglobulin levels, infectious risk, and mortality with rituximab and hypogammaglobulinemia. JAMA Netw. Open.

[24] Kimby E. (2005). Tolerability and safety of rituximab (MabThera). Cancer Treat. Rev..

[25] Xu X.T., He D.L., Tian M.X., Wu H.J., Jin X. (2022). Prognostic value of sarcopenia in patients with diffuse large B-cell lymphoma treated with R-CHOP: A systematic review and meta-analysis. Front. Nutr..

[26] Albano D., Dondi F., Ravanelli M., Tucci A., Farina D., Giubbini R., Treglia G., Bertagna F. (2022). Prognostic role of “radiological” sarcopenia in lymphoma: A systematic review. Clin. Lymphoma Myeloma Leuk..

[27] Guo J., Cai P., Li P., Cao C., Zhou J., Dong L., Yang Y., Xuan Q., Wang J., Zhang Q. (2021). Body composition as a predictor of toxicity and prognosis in patients with diffuse large B-cell lymphoma receiving R-CHOP immunochemotherapy. Curr. Oncol..

[28] Xiao D.Y., Luo S., O’Brian K., Ganti A., Riedell P., Sanfilippo K.M., Lynch R.C., Liu W., Carson K.R. (2016). Impact of sarcopenia on treatment tolerance in United States veterans with diffuse large B-cell lymphoma treated with CHOP-based chemotherapy. Am. J. Hematol..

[29] Jhaveri K., Thapa R., Ercan D., Saha A., Noble J., Singh P., Fahrmann J., Saini N., Wu R., Dennison J.B. (2025). Sarcopenia and skeletal muscle loss after CAR T-cell therapy in diffuse large B-cell lymphoma. Clin. Cancer Res..

[30] Mancuso S., Mattana M., Santoro M., Carlisi M., Buscemi S., Siragusa S. (2022). Host-related factors and cancer: Malnutrition and non-Hodgkin lymphoma. Hematol. Oncol..

[31] Lucijanić M., Huzjan Korunić R., Sedinić M., Kušec R., Pejša V. (2021). More pronounced muscle loss during immunochemotherapy is associated with worse clinical outcomes in newly diagnosed patients with diffuse large B-cell lymphoma with unfavorable features. Ther. Clin. Risk Manag..

[32] Pejša V., Prka Ž., Lucijanić M., Mitrović Z., Piršić M., Jakšić O., Ajduković R., Kušec R. (2017). Rituximab with dose-adjusted EPOCH as first-line treatment in patients with highly aggressive diffuse large B-cell lymphoma and autologous stem cell transplantation in selected patients. Croat. Med. J..

[33] Dunleavy K., Tay K., Wilson W.H. (2010). Rituximab-associated neutropenia. Semin. Hematol..

[34] Rozman S., Sonc M., Novakovic B.J. (2012). Late-onset neutropenia following primary treatment of diffuse large B-cell lymphoma with rituximab-containing therapy. Leuk. Lymphoma.

[35] Sehn L.H., Martelli M., Trněný M., Liu W., Bolen C.R., Knapp A., Sahin D., Sellam G., Vitolo U. (2020). A randomized, open-label, Phase III study of obinutuzumab or rituximab plus CHOP in patients with previously untreated diffuse large B-cell lymphoma: Final analysis of GOYA. J. Hematol. Oncol..

[36] Manz M.G., Boettcher S. (2014). Emergency granulopoiesis. Nat. Rev. Immunol..

[37] Merryman R.W., Redd R., Jeter E., Wong J.L., McHugh K., Reynolds C., Nazzaro M., Varden A., Brown J.R., Crombie J.L. (2022). Immune reconstitution following high-dose chemotherapy and autologous stem cell transplantation with or without pembrolizumab maintenance therapy in patients with lymphoma. Transplant. Cell. Ther..

[38] Compagno N., Malipiero G., Cinetto F., Agostini C. (2014). Immunoglobulin replacement therapy in secondary hypogammaglobulinemia. Front. Immunol..

